# Plant Trait Assembly Affects Superiority of Grazer's Foraging Strategies in Species-Rich Grasslands

**DOI:** 10.1371/journal.pone.0069800

**Published:** 2013-07-26

**Authors:** Jan Mládek, Pavla Mládková, Pavla Hejcmanová, Miroslav Dvorský, Vilém Pavlu, Francesco De Bello, Martin Duchoslav, Michal Hejcman, Robin J. Pakeman

**Affiliations:** 1 Department of Botany, Palacký University, Olomouc, Czech Republic; 2 Department of Ecology, Czech University of Life Sciences, Prague, Czech Republic; 3 Department of Animal Science and Food Processing in Tropics and Subtropics, Czech University of Life Sciences, Prague, Czech Republic; 4 Crop Research Institute, Prague, Czech Republic; 5 Department of Functional Ecology, Czech Academy of Sciences, Tøeboò, Czech Republic; 6 The James Hutton Institute, Aberdeen, United Kingdom; Stockholm University, Sweden

## Abstract

**Background:**

Current plant – herbivore interaction models and experiments with mammalian herbivores grazing plant monocultures show the superiority of a maximizing forage quality strategy (MFQ) over a maximizing intake strategy (MI). However, there is a lack of evidence whether grazers comply with the model predictions under field conditions.

**Methodology/Findings:**

We assessed diet selection of sheep (*Ovis aries*) using plant functional traits in productive mesic *vs.* low-productivity dry species-rich grasslands dominated by resource-exploitative *vs.* resource-conservative species respectively. Each grassland type was studied in two replicates for two years. We investigated the first grazing cycle in a set of 288 plots with a diameter of 30 cm, i.e. the size of sheep feeding station. In mesic grasslands, high plot defoliation was associated with community weighted means of leaf traits referring to high forage quality, i.e. low leaf dry matter content (LDMC) and high specific leaf area (SLA), with a high proportion of legumes and the most with high community weighted mean of forage indicator value. In contrast in dry grasslands, high community weighted mean of canopy height, an estimate of forage quantity, was the best predictor of plot defoliation. Similar differences in selection on forage quality *vs*. quantity were detected within plots. Sheep selected plants with higher forage indicator values than the plot specific community weighted mean of forage indicator value in mesic grasslands whereas taller plants were selected in dry grasslands. However, at this scale sheep avoided legumes and plants with higher SLA, preferred plants with higher LDMC while grazing plants with higher forage indicator values in mesic grasslands.

**Conclusions:**

Our findings indicate that MFQ appears superior over MI only in habitats with a predominance of resource-exploitative species. Furthermore, plant functional traits (LDMC, SLA, nitrogen fixer) seem to be helpful correlates of forage quality only at the community level.

## Introduction

The processes influencing patterns of diet selection have been brought together in optimal foraging theory which states that diet selection of a herbivore is influenced by the trade-off between the benefit of consuming a preferred diet, and the cost of handling and searching for it [1]. The diet composition selected by herbivores, therefore, usually substantially differs from the theoretically preferred diet, i.e. forage that the animals select when given a minimum of physical constraints. In heterogeneous grasslands, a herbivore's selection of the best quality components is impeded by their low abundance [2] and/or complex spatial distribution [3], [4]. Especially in species-rich grasslands herbivores must solve two opposing problems: obtaining maximum quality and sufficient quantity (see review by Hejcmanová & Mládek [5]). This implies a trade-off in decision making which operates hierarchically at several spatial and temporal scales [6], [7]. Two contrasting foraging strategies, maximizing intake (MI) or maximizing forage quality (MFQ), may be adopted by herbivores under different environmental conditions [8]–[10]. The effectiveness of these strategies has been tested in models of plant – herbivore interactions [11], [12] that each predicted the superiority of a MFQ over a MI strategy. Experiments with mammalian herbivores grazing plant monocultures [7], [13], [14] have also shown the superiority of a MFQ strategy.

Within a diverse grassland productive plant species or vegetation patches (favoring higher intake of biomass by herbivores) typically have high concentrations of fiber and low concentrations of nutrients, thus cause slower passage and digestion rates. Conversely, highly digestible plants/patches are often less productive (due to their short-living tissues) and allow only low intake rates [15]. It has been suggested that diet selection can be explained by the energy gain maximization hypothesis at all spatial scales [16]. However, the finer the scale the smaller the associated costs and benefits of selection, and the harder it is to assess them by the animal [17]. Therefore, differential defoliation of feeding stations (defined as the area available to a herbivore without moving its front feet [6]) within a paddock might be ruled by the marginal forage value which needs to be determined first by sampling [10]. On the other hand, selection of plants within a feeding station seems to be ruled by the momentary maximization hypothesis [6], which assumes that herbivores instantaneously select relatively better forage from an array of plants that it can reach without moving. When the best remaining item at the feeding station is below a certain threshold, or when the rate of forage acquisition at that station falls below that threshold, the animal moves forward, establishing a new feeding station at which diet selection proceeds again [1]. As resources are gradually depleted, herbivores must return to sampling and generate a new threshold value [18]. Therefore a herbivore's short-term selection pattern may substantially differ from the long-term pattern and, for instance, general recommendations for pasture management based on outcomes of diet selection studies performed over only a few days may be misleading.

In species-rich grasslands, the MFQ – MI dilemma has been addressed only in a few controlled experimental [9], [19] or observational [8] studies assessing diet selection in single vegetation units. Diet selection strategies have rarely been compared between communities (but see [20]), probably as a result of difficulties in assessing both quantity and quality in fine-grained, heterogeneous environments. Standing biomass and compressed sward height [21] are mainly used as indicators of forage availability (quantity). Organic matter digestibility has been highly recommended as an indication of forage quality [15], [22], but this is not easily measurable and also does not usually exactly reflect the plant's palatability for herbivores [14]. Under field conditions palatability is affected by many plant mechanical structures (e.g. thorns, hairiness, rosette leaves) which are not assessed by laboratory measurements of forage quality. An alternative method for direct comparison of diet selection across distinct vegetation units has become available recently due to the development of plant functional classifications [23] and freely accessible databases of functional trait values for common European species [24], [25]. At the species level forage quantity has been recognized as correlated with canopy height [26]. Organic matter digestibility, at least for grass species, negatively correlates with leaf dry matter content (LDMC) and positively with specific leaf area (SLA) [27]. A herbivore's selectivity for these leaf traits, which are considered the best indicators of resource exploitative *vs.* conservative strategies, remains largely untested (but see [12], [28], [29]). Forage quality is also modified by the maturity of species [14], and hence the diet selection pattern of certain herbivores is principally ruled by flowering period [15], [30]. Another informative measure of forage quality would be the forage indicator value [31], which is freely accessible for most Central-European grassland species in BiolFlor database [24] and is currently widely used in field studies [32], [33] and models of plant – animal interactions [34]. This expert-based ordinal classification of grassland species originally developed by Klapp [35] is based on information of protein and mineral biomass concentrations, leaf/stem ratio, palatability, accessibility and seasonal duration of the plant's forage value for livestock.

The principal aim of our research was to compare patterns of diet selection in two different grassland types, mesic *Arrhenatherion* and dry broad-leaved *Bromion* grasslands, using plant functional traits. The selected grassland types provide distinct levels of both forage quantity (annual biomass production 4–6 t/ha in *Arrhenatherion* and 1–3 t/ha in *Bromion* grasslands [36]) and forage quality (during May and June ∼70% organic matter digestibility in *Arrhenatherion* and ∼60% in *Bromion* [37], [38]). This distinction is connected with dominance by resource exploitative (e.g. *Dactylis glomerata*, *Poa pratensis*) *vs.* resource conservative (e.g. *Brachypodium pinnatum*, *Bromus erectus*) species [39]. Both types are widespread in Central and Western Europe and very often serve as extensive sheep pastures [38]. As sheep (*Ovis aries*) are able to select better quality components at several spatio-temporal scales [7], [13], we suppose that sheep adopt different foraging strategies in order to exploit differently allocated energy resources most efficiently. We addressed the following hypothesis: as mesic grasslands provide a sufficient amount of available biomass of high quality, sheep here selectively feed on high quality plots/plant species (adopting the MFQ strategy), in contrast, sheep grazing dry grasslands with generally low forage quantity and low quality select plots/plant species of greater biomass in order to fill their intestinal capacity and fulfill their basic metabolic requirements (adopting the MI strategy).

## Materials and Methods

### Study sites

Two distinct grassland types, mesic and dry grasslands, were selected to study grazing selectivity by sheep in the White Carpathians Mountains which are situated in the borderland between the Czech Republic and Slovakia. Within each grassland type, two independent sites were selected (Table 1). Mesic grasslands of the *Arrhenatherion elatioris* Luquet 1926 alliance included grassland ‘Mesic1’ (Brumov – Nad tunelem, 49°05′28″N, 18°01′40″E) and grassland ‘Mesic2’ (Petrùvka, 49°06′00″N, 17°49′00″E) with lower mean annual temperatures and higher mean annual precipitation than selected dry grasslands [40]. Dry grasslands of the *Bromion erecti* Koch 1926 alliance were represented by grassland ‘Dry1’ (Brumov – Klobucká, 49°05′57″N, 18°01′55″E) and grassland ‘Dry2’ (Suchovské Mlýny, 48°53′19″N, 17°33′50″E). All four sites had been unmanaged for ∼15 years prior to the start of the study, therefore tall vegetation has established in all grasslands. Extensive rotational grazing with two grazing cycles per year was applied at all study sites. Approximately 80 cross-breed sheep of Walachenschaf, Merinolandschaf, Romney and Suffolk breeds grazed a 2-ha paddock for one month at each study site. All grasslands possess haplic cambisol soils developed on tertiary flysch sediments, consisting of alternating sandstone and rock clay layers of variable thickness [38].

**Table 1:** Characteristics of sites within both grassland types, biotic parameters averaged over both years of observation.

	Mesic1	Mesic2	Dry1	Dry2
Altitude (m a.s.l.)	370	450	370	390
Aspect	NW	E	SW	SSW
Inclination (°)	19.0	15.3	17.9	18.8
MAT (°C)	6.9	6.8	7.9	7.7
MAP (mm)	800	780	760	730
Herb layer cover (%)	85.1	86.6	78.8	83.5
Moss layer cover (%)	5.7	13.1	34.1	30.9
Species richness (per site/0.07 m^2^)	66/10	62/12	66/9	65/11
CSH before grazing (cm)	8.7±2.9	6.6±2.2	10.4±2.7	8.9±2.0
CSH after grazing (cm)	10.3±4.2	6.3±2.7	11.6±3.9	8.1±2.3

Note: MAT – mean annual temperature, MAP – mean annual precipitation [40], CSH – compressed sward height measured with rising-plate meter [21]: figure behind ‘±’ standard deviation.

### Ethics statement

In all four sites our experiments were approved by private landowners, farmers and by the Administration of the White Carpathian Landscape Protected Area. All experiments were conducted in cooperation with farmers, who owned the grazing animals and supplied them regularly drinking water and mineral supplements. Sheep grazing was carried out in strict accordance with animal welfare and the recommendations stated in ‘The principles of good agricultural practice’ (Government Regulation No. 241/2004). The approval of the Animal Care and Use Committee of the Czech Ministry of the Environment was not necessary, because according to Czech law No. 246/1992 (§15: Protection of experimental animals, point 3f) permission is not needed when the acts do not cause the animal pain, suffering, distress or lasting harm.

### Experimental design and species data collection

Four 34-meter parallel transects were marked out in each grassland. Transects followed the slope upwards and were placed two meters from one another. 18 permanent circle plots situated two meters apart were established on each transect and marked with an iron nail in the soil. The circle plots were 30 cm in diameter in order to approximate the plot to the sheep's feeding station. In this rectangular grid of 72 monitoring plots we recorded the cover of the herb layer and moss layer. Further, species biomass proportions in each plot were estimated using a calibrated weight-estimate method [41]. At first, visual estimates of the absolute biomass of a species were calibrated by clipping and weighing in several training plots. When consistent estimates were attained, direct estimations of species proportions in the biomass of the studied plots were undertaken. Data collection prior to grazing was undertaken from 7 to 11 May at all sites in both years. We studied the effect of the first grazing cycle for two years (2005 and 2006) in one paddock at each site. Therefore, the rate of species defoliation was estimated using a grazed-class method [42] after one month of extensive sheep grazing for each species within each plot from 8 to 15 June. In order to avoid incorrect judgments we decided to select three broad classes of species defoliation: intact ∼0% (0–1%; because of possible insect or snail defoliation), touched ∼20% (1–40%), and eaten ∼75% (40–100%; percentage mean moved to 75% due to slightly higher frequency of severely defoliated plants) of aboveground plant biomass grazed. The scale of species defoliation was determined visually by comparison with undefoliated plants in the neighboring paddocks grazed immediately after the experimental ones. The 21 most abundant species which were common in both sites of mesic type and 21 most abundant species common in both sites of dry type are given in Appendix S1 (see Supporting Information).

Furthermore, the compressed sward height (CSH) was measured with a rising-plate meter (plate diameter 30 cm, weight 0.2 kg [21]) in each plot before and after the first grazing cycle. Comparison of CSH measured before and after grazing allowed for an estimation of the overall grazing intensity.

### Functional classification of species

The following traits (Table 2 contains their abbreviations used further in the Figures) were extracted from BiolFlor database [24] for all 139 herbaceous plant species occurring in all grasslands and years: guild (its detailed classification was converted to three broader classes – grasses: *Poaceae* family, legumes: *Fabaceae* family, forbs: all other herbaceous species), forage indicator value (ordinal scale from 1 indicating the lowest forage value to 9 indicating the highest value), onset of flowering (ordinal scale, in months); from the LEDA traitbase [25]: specific leaf area, leaf dry matter content and canopy height (distance between the highest photosynthetic tissue and the base of the plant).

**Table 2:** Plot defoliation, biomass proportions of plant taxonomic groups and plot community weighted means of traits (including their abbreviations) averaged over both sites and years within grassland type, standard deviation in parentheses.

		Grassland type			
	Unit/range	Mesic		Dry	
Plot defoliation	%	54	(18)	47	(19)
Grasses	%	45	(21)	52	(21)
Legumes	%	9	(10)	10	(11)
Forbs	%	46	(20)	38	(19)
Canopy height (Canopy)	m	0.42	(0.08)	0.41	(0.08)
Forage indicator value (Forage)	1–9	5.3	(1.1)	4.4	(0.8)
Onset of flowering (Flower)	1–12	5.5	(0.3)	5.5	(0.3)
Leaf dry matter content (LDMC)	mg g^−1^	258	(28)	293	(28)
Specific leaf area (SLA)	mm^2^ mg^−1^	21.7	(2.1)	20.4	(1.8)

### Assessment of selection of plots within grassland type

In order to analyze sheep diet selection at the scale of a paddock (between plots), plot defoliation *PD_j_* was calculated as follows:
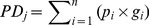
(1)where *p_i_* was the proportion of species *i* in plot *j* and *g_i_* was the rate of defoliation of species *i* in plot *j* (three classes converted into percentages– 0, 20, 75), *n* was the number of species in plot *j*. Thus, plot defoliation could range between 0 and 75%. Further, we investigated the importance of plant functional traits for plot defoliation. Many studies have made evident that functional trait values of species may be scaled up to community weighted functional properties by weighting according to relative species abundances [23]. Therefore, community weighted means *A_j_* of the above traits were calculated for each plot *j* as follows:
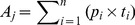
(2)where *p_i_* was the proportion of species *i* in plot *j*, *t_i_* was the trait value of species *i*, *n* corresponded to the number of species in plot *j*.

### Assessment of selection of taxonomic/functional groups of species within plots

Sheep selectivity within plots was evaluated using two different approaches: analyzing the selection of taxonomic group of species (grasses, legumes, forbs) and analyzing the selection of sum of species possessing higher trait values than the plot specific community weighted trait mean. Adopting the first approach, we looked at exclusive selection of taxonomic groups of species. This was done accordingly: biomass proportions of species in the plot as well as the proportions of species in the diet were aggregated into the following pairs: grass : non-grass, legume : non-legume, forb : non-forb. Selectivity was assessed using Jacobs' selectivity index *Di* [43] which ranges from −1 (absolute avoidance) to +1 (absolute preference).
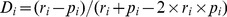
(3)where *p_i_* was the biomass proportion of grasses (or legumes or forbs) in the plot and *r_i_* was the proportion of the same group in the sheep diet in the respective plot. Before *Di* computation the *r_i_* was calculated separately for each plant species *i* using individual estimates of species defoliation rate *g_i_* as follows:
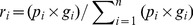
(4)where *n* was the number of species in the plot.

The second approach was based upon the concept of momentary maximization at a feeding station [6]. This concept stimulated us to analyze which plant traits correlate to the sheep's perception of their feeding station (circle plot 30 cm in diameter) and hence are likely to drive its selection. According to the momentary maximization hypothesis a herbivore instantaneously selects relatively better forage from an array of plants that it can reach without moving, thus we computed community weighted trait mean at each plot (plot specific) and compared the trait values of all plant species present in the plot with this plot specific community weighted trait mean. For example, all plant species taller than the community weighted mean of canopy height in plot number 1 were put into one group and Jacob's selectivity for this group was calculated (this procedure was applied also in plot number 2, 3 … 288). In general, selectivity was calculated for the group of species possessing higher trait values *t_i_* than the plot specific community weighted mean *A_j_*. First, proportions *p_i_* of *k* species in plot *j* with higher trait values than plot specific community weighted mean *A_j_* were summed:
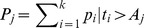
(5)Further, diet proportions *r_i_* of *k* species in plot *j* were summed as follows:
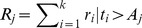
(6)Jacobs' selectivity from proportions *P* and *R* in plot *j* was calculated using equation 3.

### Statistical analysis

As our dataset involved random effects, statistical analyzes were done with linear mixed effects models [44] where normality and homoscedasticity of residuals were always checked. First, we analyzed plot defoliation across both grassland types and subsequently performed the same analysis of plot defoliation for mesic and dry grassland types separately. Plot defoliation was the dependent variable, community weighted means of quantitative traits and the biomass proportions of taxonomic groups were considered as separate fixed effects while site code in a given year of observation as the only one random effect, because there was no spatial or temporal autocorrelation of plot defoliation at any site.

Analyzing diet selection within plots, sheep selectivity for plant traits was compared for mesic and dry grasslands using linear mixed effect models where Jacobs' D was the dependent variable, grassland type (mesic and dry) was treated as a fixed effect, while site code in a given year of observation was used as a random effect. Jacobs' selectivity for a particular trait was considered significantly positive/negative if the confidence interval did not involve a zero value [20]. Quantitative comparison of Jacobs' D values from different samples was appropriate as only two food types (e.g. grass *vs.* non-grass; group of species with higher LDMC than plot specific community weighted mean *vs.* group of species with lower LDMC than plot specific community weighted mean) were considered [45]. Linear mixed effects models were performed with R 2.10.1 software (www.r-project.org) using the ‘nlme package’ [46].

## Results

### Functional characteristics of grassland types and comparison of grazing intensity

The investigated grasslands showed similar abiotic characteristics (Table 1), but both dry grassland sites had higher mean annual temperatures, lower precipitation rates and a south-western aspect. Moreover, in the dry grasslands we recorded a lower cover for the herb layer, a higher cover of moss, lower community weighted means of forage indicator value and SLA, and a higher community weighted mean of LDMC (Table 2).

Comparison of compressed sward height before and after grazing (Table 1) showed that within each grassland type there was one site with greater grazing intensity (decreased average CSH after grazing in Mesic2 and Dry2) and one site with more extensive grazing (increased CSH in Mesic1 and Dry1). Therefore, patterns of diet selection in grassland types cannot be a product of grazing intensity.

### Diet selection between plots

**Figure 1:**
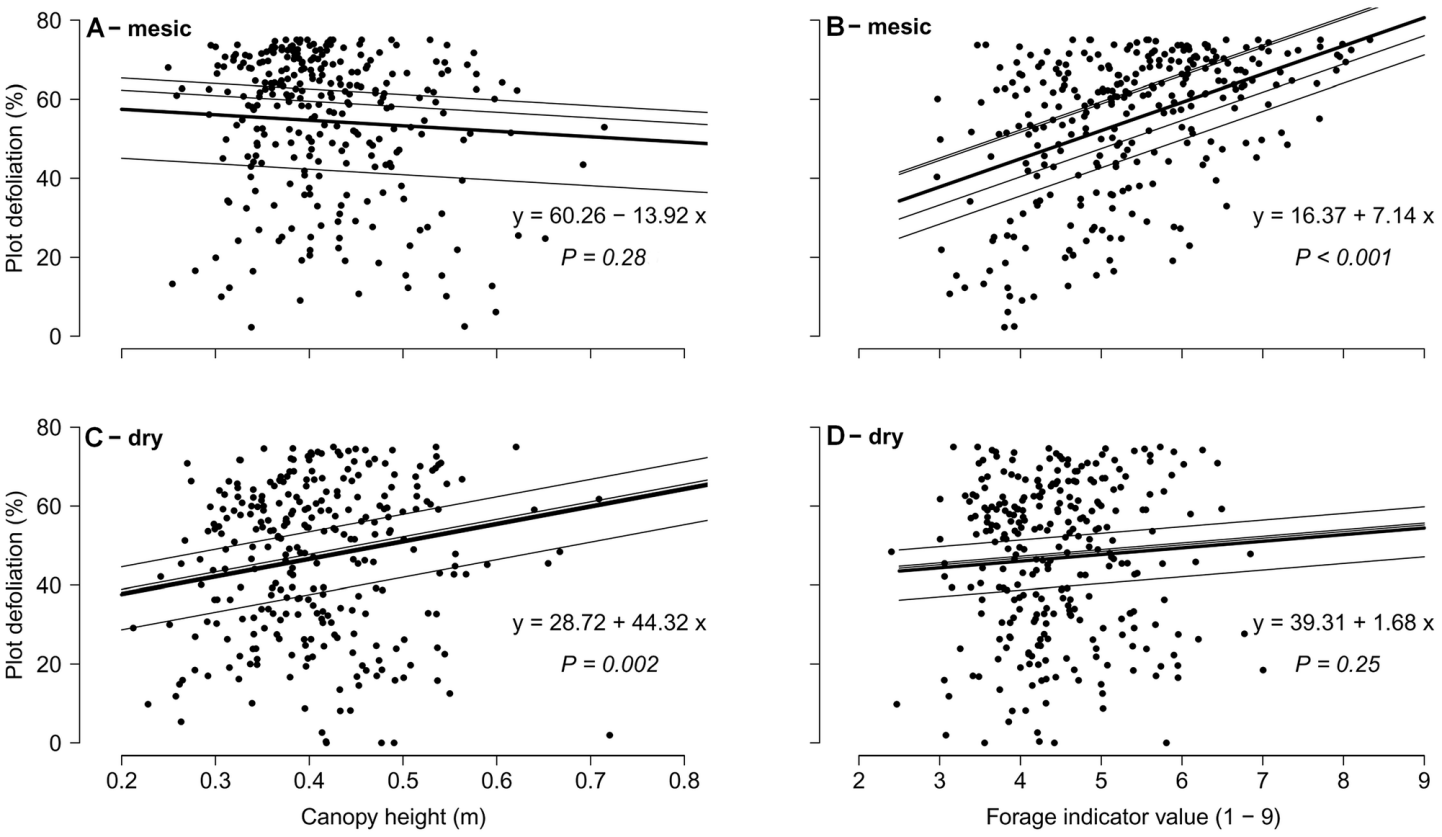
Relationship between plot defoliation and community weighted means of traits in mesic (A–B) and dry (C–D) grasslands. The four thin regression lines on each graph represent models for both sites in both years of observation (all levels of random effect), thick line (overlapping some thin lines) and its formula belongs to final mixed effect model.

At first, plot defoliation was compared across both grassland types using a mixed effect model where grassland type, community weighted means of quantitative traits, biomass proportions of grasses and legumes and their first order interactions with grassland type were used as fixed effects (AIC = 4890.03). Simplification of the model using the Maximum Likelihood method [44] led to the final model (AIC = 4852.90) where grassland type, canopy height, forage indicator value, onset of flowering, LDMC, the proportion of grasses and only two interactions with grassland type remained (type : canopy height, *P*<0.001; type : forage indicator value, *P = *0.003). Significant interactions of both canopy height and forage indicator value with the grassland type were evidence that sheep employed different quantity/quality strategies when grazing mesic and dry grasslands. The considerable effect of grassland type on diet selection is clear from the different slopes of the regression lines (Fig. 1). Canopy height was not important for plot defoliation in mesic but increased plot defoliation in the dry grassland type. Forage indicator value enhanced plot defoliation in mesic, but was not important in dry grasslands.

Inspecting plot defoliation with separate mixed effect models (Table 3) we revealed that different community weighted means of traits affected the pattern of sheep diet selection in mesic and dry grasslands. In the mesic type, the most pronounced was the positive effect of forage indicator value, followed by the negative effect of LDMC and positive effects of SLA and the proportion of legumes. In the dry type, plot defoliation was promoted the most by greater canopy height, then by the proportion of grasses and later onset of flowering whereas a high proportion of forbs reduced sheep grazing on a plot.

**Table 3:** Linear mixed effect models where plot defoliation was dependent variable and every biomass proportion of taxonomic group or community weighted mean of quantitative trait was treated in the separate model and considered as fixed effect, while site code in a given year as random effect (degrees of freedom 1, 283).

	Mesic grasslands			Dry grasslands		
	*Effect*	*F*	*P-value*	*Effect*	*F*	*P-value*
*Biomass proportions of taxonomic groups*						
Grasses		0.01	0.94	+	7.07	0.0083
Legumes	+	10.44	0.0014		0.30	0.59
Forbs		2.44	0.12	−	8.25	0.0044
*Community weighted means of quantitative traits*						
Canopy		1.15	0.28	+	9.69	0.002
Forage	+	75.77	<0.001		1.30	0.25
Flower		1.19	0.28	+	4.34	0.038
LDMC	−	16.49	<0.001		0.01	0.94
SLA	+	15.43	<0.001		0.02	0.90

### Diet selection within plots

**Figure 2:**
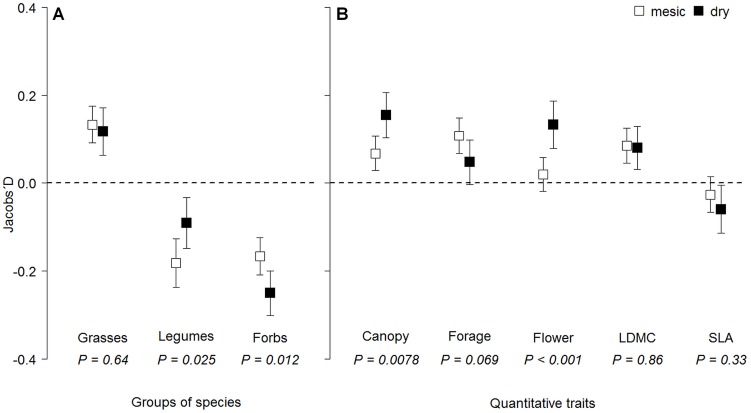
Sheep selectivity (Jacobs' D) within plots in mesic and dry grasslands. Selectivity for (A) taxonomic groups of species; (B) sum of species possessing higher trait values than plot specific community weighted trait mean. Selectivity was evaluated with Jacobs' D selectivity index ranging from −1 to 0 (avoidance) and from 0 to +1 (preference). Squares show means with 95% confidence interval (CI). Selectivity for a particular group/trait was considered significantly positive/negative if CI did not involve a zero value.

Evaluating the selection of the taxonomic groups of species (Fig. 2A) we detected positive selection of grasses while avoidance of legumes (more pronounced in mesic grasslands) and forbs (more pronounced in dry grasslands).

Furthermore, consistently with diet selection between plots the within plot analyzes of functional traits showed a clear trade-off in selection on quantity in dry *vs.* quality in mesic grasslands (Fig. 2B). In the dry grasslands, with regard to the plot specific community weighted mean, sheep selected plant species with greater canopy height but not with higher forage indicator value (confidence interval involved zero). In the mesic grasslands, sheep exhibited a stronger positive selection of species with higher forage indicator value compared to dry grasslands (marginally significant *P = *0.069), and also selected species with greater canopy height but substantially less strongly than in the dry grasslands. Further, correspondingly to the pattern between plots, sheep selected plant species with a later onset of flowering only in the dry grasslands. Contrary to the selection between plots we found unexpectedly positive selection of species with higher LDMC and rather negative selection of species with higher SLA within plots in both mesic and dry grasslands.

## Discussion

Our results indicate how foraging strategies of herbivores change according to the plant trait assembly, i.e. community weighted means of plant traits which are known to correlate with the quantity and quality of available resources. As we had assumed, the selected mesic grasslands were differentiated by higher community weighted means of forage indicator value and SLA, and a lower community weighted mean of LDMC (Table 2) – i.e. indicators of higher forage quality [27], [47]. The supposed higher forage quantity (availability) in mesic grasslands was not due to vegetation height, as the community weighted mean of canopy height was in a similar range in both grassland types, probably because all sites had been unmanaged for a long time and tall species were favored. The higher quantity of forage in mesic grasslands was therefore due to higher vegetation density. We had assumed that different biomass would be well reflected by measurements of compressed sward height [21], but this was not the case because more rigid stems in dry grasslands probably magnified the measurements. Hence, the higher availability of forage for sheep in mesic grasslands was indicated by a higher cover of the herb layer. Moreover in dry grasslands, we recorded an average cover of the moss layer of almost three times higher than that in the mesic grasslands (Table 1), and high bryophyte biomass has been shown to be a good indicator of low herb layer density [48]. Our assumptions were met; sheep appeared to use the MFQ strategy in the mesic grasslands whilst they used the MI strategy in dry grasslands consistently across different spatial scales. This finding corresponds to the conclusion that smaller-bodied ‘large herbivores’ such as sheep are able to perceive and exhibit selectivity at multiple scales simultaneously [7]. Sheep exhibited a consistent pattern of selection at the community level (selection between plots) and at the plant level (selection within plots, i.e. feeding stations). Our findings might seem contradictory to the study by Thomas et al. [14], who reported higher sheep preference for highly nutritious plants when the vegetation was of low quality. However, their conclusions are based on an experiment in which sheep had free access to plant monocultures of equal size, so that the sheep grazing pattern was not constrained by low quantity and/or dispersed distribution of preferred plant species. Therefore these different conclusions are the consequence of preference and selection concepts, and direct comparison of their outputs deserves further attention (up to now done only for invertebrate herbivores [12]).

### Diet selection between plots – community level

As mesic grasslands provided forage of relatively high quantity sheep should benefit from a larger selection on quality. Indeed, sheep selected plots with higher forage indicator value, higher SLA, lower LDMC and lower canopy height (Table 3) which is in line with results of a study from fertile Argentinean steppe [28] that related community weighted means of traits and sheep selectivity. This foraging strategy corresponds to predictions from the dynamic model by Hutchings and Gordon [11] and empirical results by García et al. [13], who both concluded that the MFQ strategy is the most efficient strategy for grazing throughout the season whatever the stocking rate. In dry grasslands, however, it seems that the cost paid by sheep for searching and/or handling of higher quality plots was not compensated by respective energy gain. This was probably because of the low forage quality (as shown by the low forage indicator value). In addition, low levels of plant biomass made it rather more efficient for sheep to maximize energy gain by selecting plots of higher quantity – i.e. plots with greater canopy height (cf. [2], [9], [49]). Sheep diet selection was associated with high proportion of grasses but also with later onset of flowering in dry grasslands (Table 3) which may indicate that beside a prime focus on forage quantity, sheep tended to maintain the quality of their diet as the later flowering species often exhibit better nutritional value in spring than early flowering species [50].

### Diet selection within plots – plant level

Selection in a heterogeneous environment, where each species occurs with different abundance, should be evaluated with a suitable selectivity index. We chose Jacobs' selectivity index rather than other indices for its low sensitivity to variations in relative species abundances [10], [45]. Grasses were selected within plots instead of dicotyledons (both legumes and forbs) in both grassland types (Fig. 2A), which might be attributed to the period of grazing as several studies of sheep diet selection recorded that the initial spring preference for grasses shifted to dicotyledons in summer [8], [51]. Probably, during our first grazing cycle (7–11 May until 8–15 June), the grass biomass had sufficient digestibility and sheep were not yet forced to choose dicotyledons, which maintain their nutritive value longer in the season [52], but may cause digestive problems due to secondary metabolites [22].

Many studies have shown a preference of sheep for legumes when grown with grasses [2]. Although sheep preferred plots with a high proportion of legumes (in mesic type), this group of plants was avoided within plots. Such a discrepancy can be explained by legume's ability to enrich soil with nitrogen and thus support growth and leaf nitrogen concentration of neighboring species [53]. Thus, sheep selectivity of plots with a high proportion of legumes might be rather caused by this indirect effect than by forage quality of diverse legumes in semi-natural grasslands which is on average much lower than that of *Trifolium repens*, commonly used as the legume in diet selection studies.

### Assessment of product of maximizations within plot – significance of functional traits

The momentary maximization hypothesis [6] assumes that a grazing animal selects a diet from a sensorially defined array of plants that it can reach without moving (i.e. within the feeding station). Although we did not observe the instantaneous decisions of animals within the feeding station, we were interested in the final effect of several momentary decisions made by different animals during the first grazing cycle in our permanent plots (30 cm in diameter). We supposed that sheep are able to perceive an ‘average forage value’ and select relatively within a plot. In order to assess a product of all within-plot momentary maximizations, we performed an analysis of selection for the sum of species possessing a higher trait value than the plot specific community weighted mean (see Methods). The results revealed the importance of plant traits for relative within-plot selection over a one month period. Even in this aspect of selection, sheep applied the MFQ strategy (indicated by forage indicator value) in mesic grasslands and the MI strategy (canopy height) in dry grasslands (Fig. 2B). Moreover, within plots sheep also favored later flowering species in dry grasslands, and thus confirmed that early phenological stages of plants are preferably grazed by herbivores as these should have higher nutritional quality in terms of available energy and protein [30].

As for grasses digestibility negatively correlates with LDMC and positively with SLA [27], positive selection of plant species with higher SLA or lower LDMC might be expected [29]. However, reverse patterns of selection were found within plots (Fig. 2B). Such a result could be attributed to the high proportion of the grassland biomass created by dicotyledons (55 and 47% of the total in mesic and dry grasslands, respectively). Moreover, by analyzing species pools at each site we found no relationships between species forage indicator value and SLA or LDMC. Consistent with our results, Cingolani et al. [28] reported that SLA is not a good predictor of forage quality at the plant level as sheep surprisingly selected plants with tougher leaves. This is in line with positive selection of plants with higher LDMC within plots in both our grassland types, and corresponded to positive selection of grasses instead of dicotyledons (Fig. 2A) as grasses occurring within the grassland patch generally possess higher LDMC than their surrounding dicotyledonous neighbors [54].

### Conclusions

We have shown here, for the first time to our knowledge, that foraging strategies of selective mammalian grazers such as sheep might be modulated by plant trait assembly. Although studied species-rich grasslands (on average 65 species per site) shared many plant species (Appendix S1), the predominance of resource-exploitative or resource-conservative plants led sheep to adopt different foraging strategies. When forage is abundant and offers a choice of highly nutritious species, the MFQ strategy appears to be the most favorable. On the other hand, if the sward consists mainly of species of low forage indicator value the MI strategy seems to be more efficient in maximizing energy gain. Such contrast in herbivore exploitation of abiotically divergent habitats may be likened to differences between the MFQ strategy adopted in spring and the MI strategy being valid in autumn and winter [8], [10]. These temporal alterations of foraging strategies are related to high food availability and high quality in spring in contrast to low availability and low quality late in the season. Such temporal differences are analogical to the differences in forage characteristics between mesic *vs.* dry grasslands which are induced by divergent environmental conditions. The point where the shift between MFQ and MI strategies occurs will likely vary depending on the size of the herbivore: smaller herbivores will be capable of continuing longer with the MFQ strategy as overall forage quality declines (cf. [7]). We advocate performing more studies of diet selection in semi-natural grasslands differing in plant trait assembly, this could provide better understanding of various grazer effects under different environmental conditions, which has puzzled ecologists for a long time [28], [50], [55], [56] and remains to be fully resolved.

## Supporting Information

Appendix S1 **Frequency and biomass proportion of the most abundant plant species.** Note: Original dataset is not deposited in publicly available resources as this is not required in the field of ecology, but we are willing to provide it to any scientist who will be interested in.
(DOC)
