# Supplementary material for: Plant Trait Assembly Affects Superiority of Grazer's Foraging Strategies in Species-Rich Grasslands
Source: PLoS One. 2013 Jul 26;8(7):e69800. doi: 10.1371/journal.pone.0069800 (PMC3724891; doi:10.1371/journal.pone.0069800)
Supplement: Appendix S1 — Frequency and biomass proportion of the most abundant plant species. Note: Original dataset is not deposited in publicly available resources as this is not required in the field of ecology, but we are willing to provide it to any scientist who will be interested in. (DOC) [file pone.0069800.s001.doc]

Appendix S1. Frequency of occurrence (frequency = % of occupied plots) and average biomass proportion (biomass) in the plots before grazing for the 21 most abundant species in both sites of mesic grasslands and for the 21 most abundant species in both sites of dry grasslands.

| Grassland type | Mesic | |  | Dry | |
| --- | --- | --- | --- | --- | --- |
|  | frequency | biomass |  | frequency | biomass |
| Grasses |  |  |  |  |  |
| *Agrostis capillaris* | 19 | 0.01 |  |  |  |
| *Brachypodium pinnatum* |  |  |  | 55 | 0.16 |
| *Bromus erectus* |  |  |  | 59 | 0.16 |
| *Dactylis glomerata* | 35 | 0.03 |  | 15 | 0.01 |
| *Elytrigia repens* | 40 | 0.04 |  |  |  |
| *Festuca pratensis* | 36 | 0.04 |  |  |  |
| *Festuca rubra* | 46 | 0.07 |  |  |  |
| *Festuca rupicola* | 25 | 0.06 |  | 40 | 0.05 |
| *Poa pratensis* | 78 | 0.08 |  | 44 | 0.07 |
| *Trisetum flavescens* | 58 | 0.03 |  | 13 | 0.01 |
|  |  |  |  |  |  |
| Legumes |  |  |  |  |  |
| *Lotus corniculatus* |  |  |  | 16 | 0.01 |
| *Medicago falcata* | 42 | 0.04 |  | 14 | 0.01 |
| *Securigera varia* |  |  |  | 23 | 0.02 |
| *Trifolium medium* | 17 | 0.01 |  |  |  |
|  |  |  |  |  |  |
| Forbs |  |  |  |  |  |
| *Agrimonia eupatoria* |  |  |  | 8 | 0.01 |
| *Achillea millefolium* | 67 | 0.11 |  | 42 | 0.03 |
| *Carex caryophyllea* |  |  |  | 10 | 0.01 |
| *Carex flacca* |  |  |  | 20 | 0.01 |
| *Carex tomentosa* | 19 | 0.03 |  |  |  |
| *Centaurea jacea* | 31 | 0.04 |  | 13 | 0.01 |
| *Convolvulus arvensis* | 23 | 0.01 |  |  |  |
| *Crepis biennis* |  |  |  | 10 | 0.01 |
| *Daucus carota* | 23 | 0.01 |  |  |  |
| *Fragaria viridis* | 34 | 0.02 |  | 47 | 0.03 |
| *Galium verum* |  |  |  | 55 | 0.05 |
| *Potentilla reptans* | 28 | 0.02 |  |  |  |
| *Salvia pratensis* | 7 | 0.01 |  |  |  |
| *Taraxacum* sect. *Ruderalia* | 55 | 0.03 |  | 21 | 0.01 |
| *Teucrium chamaedrys* |  |  |  | 6 | 0.01 |
| *Veronica chamaedrys* | 21 | 0.01 |  | 19 | 0.01 |
| *Viola hirta* | 27 | 0.01 |  | 34 | 0.01 |

Plant names follow nomenclature: Kubát K, Hrouda L, Chrtek J, Kaplan Z, Kirschner J, Štěpánek J (2002) Key to the Flora of the Czech Republic. Praha: Academia.
